# Role of Complement Component 9 in Bone Health: Causal Evidence in Humans and Mechanistic Studies in Mice

**DOI:** 10.1007/s00223-026-01515-9

**Published:** 2026-04-02

**Authors:** Claes Ohlsson, Anna E. Törnqvist, Karin H. Nilsson, Maria Nethander, Jianyao Wu, Lei Li, Antti Koskela, Sofia Movérare-Skrtic

**Affiliations:** 1 Department of Internal Medicine and Clinical Nutrition, Institute of Medicine, Sahlgrenska Osteoporosis Centre, Centre for Bone and Arthritis Research, Sahlgrenska Academy at University of Gothenburg, Sahlgrenska University Hospital, Vita Stråket 11, 41345 Göteborg, Sweden; 2 Unit of Clinical Pharmacology, Department of Pharmaceuticals, Sahlgrenska University Hospital, Gothenburg, Region Västra Götaland Sweden; 3 Department of Anatomy and Cell Biology, Faculty of Medicine, Institute of Cancer Research and Translational Medicine, University of Oulu, Oulu, Finland

**Keywords:** Complement system, Fractures, Bone mineral density, Mendelian randomization, Mouse model

## Abstract

Complement component 9 (C9) is the main pore-forming protein of the terminal membrane attack complex (MAC) in the complement system. The MAC forms a lytic pore in the membrane of target cells and is a key effector of the innate immune system. Excessive complement activation has been implicated in chronic inflammation, which is a well-established risk factor for increased fracture susceptibility. Although other components of the complement system have been linked to bone mass regulation, the role of C9 is unknown. The aim of the present study was to determine the role of C9 for bone health. To determine the role of circulating, mainly liver-derived, C9 in humans, we conducted Mendelian randomization (MR) using a single-SNP instrument and performed genetic colocalization analyses. Genetically predicted higher circulating C9 levels were causally associated with increased risk of forearm fractures and fractures at any bone site. Colocalization analyses supported these findings. Mechanistic studies were performed in mice. As expected, high C9 expression was observed in the liver, while the expression was low in cortical bone and no expression was observed in trabecular bone. Adult female mice with global C9 deletion (*C9*^−/−^) exhibited lower vertebral trabecular bone volume fraction due to a decreased trabecular number, but they were partly protected against ovariectomy-induced trabecular bone loss. No skeletal phenotype was observed in the femur or in male *C9*^−/−^ mice. In conclusion, high genetically determined circulating C9 is causally linked to increased fracture risk in humans. In mice, global inactivation of *C9*^−/−^ affects vertebral trabecular bone in a sex-specific and context-dependent manner.

**Supplementary Information:**

The online version contains supplementary material available at 10.1007/s00223-026-01515-9.

## Introduction

The complement system, a key component of the innate immune defense, plays a central role in host protection. Most of the proteins of the complement system, including complement component 9 (C9), are synthesized in the liver and released into the circulation, although production of complement components has also been found locally in other tissues [1]. Activation of the complement system occurs via three different pathways: the classical, lectin, and alternative pathways, which all converge at the C3 level leading to the subsequent formation of C5b [2]. The terminal membrane attack complex (MAC) of the complement system consists of C5b, C6, C7, and C8, and multiple C9 molecules. C9 is the main pore-forming protein of the MAC, which forms a lytic pore in the membrane of the target cells and causes cell lysis. Recent work shows that osteocytes sense high mechanical loads via transient plasma membrane disruptions (PMDs), and that membrane fragility or defective PMD repair impairs mechanotransduction and survival. Disruption of β2‑spectrin (Sptbn1) or Prkd1‑mediated repair increases PMD burden and osteocyte death, a mechanistic context directly relevant to MAC-mediated pore formation in bone cells [3, 4].

Autologous cells are protected from the complement cascade by membrane-bound inhibitors, such as CD59, which specifically prevents the formation of the C9 lytic pore [2, 5]. However, apoptotic and necrotic autologous cells rapidly lose the membrane-bound complement inhibitors, leading them susceptible to MAC formation and subsequent lysis [6].

Given the complements system’s established role in immune defense and its association with chronic inflammation, recent studies have increasingly focused on its impact on bone health. Complement proteins have been shown to be involved in bone development and bone homeostasis [1, 7]. Moreover, excessive activation of the complement system has been implicated in chronic inflammation, a well-established risk factor for bone loss and increased fracture susceptibility [8–10]. High circulating levels of specific components such as C7 and C9 have been associated with accelerated bone loss in men [11]. In addition, high circulating C7 levels have been associated with increased risk of hip fractures [11]. Evidence from mouse models lacking various complement components further supports these roles. For instance, mice deficient of C3 exhibited increased trabecular bone and were protected against ovariectomy-induced trabecular bone loss in the long bones compared to wild type (WT) mice [12, 13]. In contrast, C6-deficient male mice displayed lower vertebral trabecular bone mass and reduced appendicular bone strength compared to WT mice [14]. Studies of CD59-deficient mice have reported variable skeletal phenotypes. One study demonstrated decreased cortical bone mineral density (BMD) in long bones of adult mice compared to WT mice, whereas old CD59-deficient mice exhibited increased cortical BMD compared to WT mice [15]. Adult male CD59-deficient mice also displayed increased cortical and trabecular bone volume and femur length [15]. Another study, however, reported no skeletal phenotype in male CD59-deficient mice [14]. Together, these findings suggest that the complement system contributes to bone homeostasis and ovariectomy-induced bone loss. However, the specific role of C9, the terminal pore-forming component of the MAC, in skeletal regulation remains unclear. Integrating human genetics with mechanistic bone studies aligns with modern preclinical skeletal safety strategies, which emphasize human‑relevant evidence to strengthen causal inference. As highlighted by Tuladhar et al. [16] combining genetic data with targeted in vivo models improves prediction of skeletal risks and enhances translational relevance. To address this, we investigated the role of C9 in bone health by integrating human Mendelian randomization data with mechanistic studies in mice.

## Materials and Methods

### Animals

Animal care was in accordance with institutional guidelines, which complied with the ARRIVE guidelines. All applicable international, national, and institutional guidelines for the care and use of animals were followed. All procedures performed were approved by the Ethics Committee in Gothenburg, Västra Götaland (Permit Number 5829/2023), and the care of the animals was in compliance with all relevant ethical regulations for animal research. The mice were housed together in a standard animal facility under controlled temperature (22 °C) and photo periods (12 h of light, 12 h of darkness) with free access to water and food pellets (Teklad diet 2016, Envigo, Indianapolis, IN, USA).

### C9 Deficient Mice

The mouse strain used for this research project, C57BL/6N-C9^tm1.1(KOMP)Vlcg^/JMmucd, RRID:MMRRC_046855-UCD, was obtained from the Mutant Mouse Resource and Research Center (MMRRC) at University of California at Davis, an NIH-funded strain repository, and was donated to the MMRRC by The KOMP Repository, University of California, Davis; Originating from Stephen Murray, The Jackson Laboratory [17]. Heterozygous C57BL/6N-C9^tm1.1(KOMP)Vlcg^/JMmucd female mice (*C9*^+/−^) were crossed with WT male mice purchased from Taconic (C57BL/6NTac). Subsequently, only *C9*^+/−^ female and male mice were used for breeding. All mice used in this study were either* C9*^−/−^ or WT littermates. The mice were genotyped using the following primers: mutant forward 5′- ACTTGCTTTAAAAAACCTCCCACAC-3′, mutant reverse 5′- ACTCTCTACAATGAGGCCCCAAATCC-3′, WT forward 5′- GTCAATGCACAGATGCCAATACC-3′, and WT reverse 5′- ACCGTTTCTTTTTCCCTCTGATCC-3′.

At 13 weeks of age, mice underwent either ovariectomy/orchidectomy or sham surgery, and the mice were terminated at 17 weeks of age (n = 6–10 per group). Before surgery all animals received analgesics (Metacam, Boehringer Ingelheim Animal Health, Ingelheim am Rhein, Germany), and all surgery was performed under isoflurane inhalation anesthesia. All efforts were made to minimize suffering. At termination, blood was collected from the axillary vein under anesthesia with Ketador/Dexdomitor (Richter Pharma, Wels, Austria/Orion Pharma, Espoo, Finland), and the mice were subsequently euthanized by cervical dislocation. Soft tissues were dissected and weighed. Liver was snap-frozen in liquid nitrogen and subsequently stored at − 80 °C. One femur and lumbar vertebrae 4 and 5 (L4 and L5) were dissected, fixed for 2 days in 4% formaldehyde, and then stored in ethanol (70% v/v) until further use.

### Quantitative Real-Time PCR Analyses of Mouse Tissue

Twelve-week-old female C57BL/6N WT mice were used to quantify *C9* expression in liver, kidney, spleen, lung, muscle, heart, hypothalamus, thymus, uterus, retroperitoneal fat, gonadal fat, brown fat, and bone (n = 6). To preserve RNA integrity, cortical bone (tibial shafts) and trabecular‑rich bone (vertebral bodies) were dissected immediately after sacrifice and stored in RNAlater (76106, Qiagen). Total RNA from liver, kidney, spleen, lung, muscle, heart, hypothalamus, thymus, and uterus was isolated using the RNeasy Mini Kit (74116, Qiagen). Due to the low RNA yield and high mineralization of skeletal tissue, RNA from cortical bone (flushed tibial shafts) and trabecular‑rich bone (vertebral bodies) was extracted using TRIzol Reagent (15596018, Thermo Fisher Scientific) combined with mechanical homogenization in a TissueLyser (Qiagen) using stainless‑steel beads, followed by column purification with the RNeasy Mini Kit (74116, Qiagen). This two-step protocol was also used to extract total RNA from retroperitoneal fat, gonadal fat, and brown fat.

*C9* expression in liver from 17-week-old sham-operated WT and *C9*^−/−^ mice (n = 8) was quantified by isolating total RNA using the RNeasy Mini Kit (74116, Qiagen). The RNA was reversed transcribed into cDNA (4368814, Applied Biosystems), and quantitative real-time PCR (qPCR) analyses were performed using the StepOnePlus Real-Time PCR System (Thermo Fisher Scientific). *C9* expression was assessed using the TaqMan Assay-on-Demand primer and probe set Mm00442739_m1 (Thermo Fisher Scientific). The expression was normalized to 18S ribosomal subunit (4310893E; Thermo Fischer Scientific). The relative gene expression was calculated using the 2^−∆∆Ct^ method. The detection limit for C9 expression was defined as a Ct value ≥ 36. Ct values at or above this threshold were considered below the limit of detection.

### Serum Measurements

The bone resorption marker collagen type I C-terminal telopeptides (CTX-I) and bone formation marker procollagen type I N-terminal propeptide (P1NP) were measured in serum using competitive enzyme immunoassay (EIA) kits (Immunodiagnostics Systems, Herlev, Denmark), according to manufacturer’s directives.

### Analyses of Femur by Peripheral Quantitative Computed Tomography (pQCT)

Peripheral quantitative computed tomography (pQCT) scans were performed using the XCT Research M (version 4.5B; Norland, Fort Atkinson, USA) operating at a resolution of 70 µm, as described previously [18, 19]. In the femur, cortical bone parameters were analyzed in the mid-diaphyseal region at a distance proximal from the distal growth plate corresponding to 36% of the total length of the femur. The scan for trabecular bone parameters was positioned in the metaphysis of the femur at a distance proximal from the distal growth plate corresponding to 3% of the total length of the femur. The trabecular bone region was defined as the inner 45% of the total cross-sectional area.

### Analyses of Axial Bone by Micro-CT (µCT)

High-resolution microcomputed tomography (µCT) analyses were performed on L4 vertebrae using the Skyscan 1275 system (Bruker MicroCT, Kontich, Belgium), with an X-ray tube voltage of 40 kV and a current of 200 µA. The angular rotation was set at 180°, with an angular increment of 0.40°. Voxel size was isotropically maintained at 7 µm. Analysis of cortical and trabecular bone parameters of L4 were initiated 7 µm caudal of the lower end of the pedicles, extending a further longitudinal distance of approximately 245 µm in the caudal direction. The region of interest for trabecular bone was manually delineated and analyzed using CTAn software (Bruker).

### Dynamic Histomorphometry

For dynamic histomorphometric analysis, mice received intraperitoneal injections of calcein (C0875, Merck GmbH) seven days before euthanasia and alizarin (A3882, Merck GmbH) one day prior to euthanasia. Following dissection, L4 vertebrae were fixed in 4% formaldehyde for 48 h, dehydrated through a graded ethanol series, incubated overnight in 30% sucrose, and subsequently embedded in OCT (optimal cutting temperature) compound. The embedded vertebrae were then longitudinally sectioned through the central region of the vertebral body using Kawamoto’s film method (2020) with Cryofilm type 3C (16UF) to obtain 10 µm cryosections [20]. Fluorescent images were acquired using a Nikon spinning-disk microscope. Histomorphometric parameters were quantified using the OsteoMeasure7 system (OsteoMetrics) in accordance with the guidelines of the American Society for Bone and Mineral Research (ASBMR) [21].

### Mechanical Strength

The mechanical strength of intact L5 was tested by axially loading the vertebra, using a mechanical testing machine (Instron 3366, Instron), as previously described [22]. Briefly, a press head of 2 mm in diameter was loaded onto L5 at a speed of 0.155 mm/s. The maximal load at failure (F_max_ (N)) was calculated by a custom-made Excel macro, using the computer-recorded load deformation raw data curves produced by Bluehill 2 software v2.6 (Instron).

### Statistical Analysis

Mouse data are presented as scatter plots, with horizontal lines indicating the mean and vertical bars representing the standard error of the mean (SEM), or presented in tables expressed as mean ± SEM. For comparison of two groups, Student’s t-test was used. To evaluate the effect of castration, genotype, and their interaction (castration by genotype), a two-way ANOVA was performed using GraphPad Prism (version 10.6.0). When the genotype-by-ovariectomy interaction was significant, we examined it using pairwise comparisons (ovariectomy vs. sham within each genotype; *C9*^−/−^ vs. WT within each surgery condition) and applied Holm–Šidák post hoc adjustments. Effect sizes were expressed as percentage changes relative to the appropriate reference group mean (e.g., ovariectomy vs. sham within each genotype; *C9*^−/−^ vs. WT within each surgery condition). In all analyses, *P* < 0.05 was considered statistically significant.

### Mendelian Randomization (MR)

To evaluate if C9 is causally associated with fracture risk, we used previously published publicly available GWAS summary statistics for circulating proteins [23], forearm fracture [24], and fracture at any bone site [25]. pQTLs for C9 were primarily identified using GWAS summary statistics of circulating proteins measured with the aptamer based SomaScan proteomic platform [23]. pQTLs were considered valid instrumental variables if they were cis-pQTLs with a minor allele frequency above 1%. To ensure independence, LD-pruning was performed using LDlink (https://ldlink.nih.gov/) with an r^2^ threshold of 0.01, keeping the most significant pQTLs. One SNP, rs835703, fulfilled these criteria and was harmonized to have the same effect allele in both protein and outcome data. The F-statistic for rs835703 (101.6) indicated a low risk of weak instrument bias. In sensitivity analyses, we used GWAS summary statistics of circulating proteins measured with the independent immunoassay-based Olink platform [23], applying the same criteria for instrument validity. One SNP, rs700233, fulfilled the criteria and was harmonized with the outcome data (F-statistic 181.5). MR analyses were performed using the mr_ivw function in the MendelianRandomization R-package, which uses the Wald ratio when only one instrumental variable is available.

### Colocalization

Colocalization analyses between C9 and forearm fracture or fracture at any bone site were performed using PWCoCo (https://github.com/jwr-git/pwcoco) with reference data from 10,000 randomly selected unrelated participants from UK Biobank. PWCoCo implements conditional analyses from GCTA-COJO [26, 27] and colocalization analyses from the R coloc-package [28] to allow for unbiased colocalization evidence. The initial analysis is performed on unconditional summary statistics for both traits. When there is evidence of colocalization on unconditional summary statistics (PP.H4 ≥ 0.8), no conditional analyses are performed. When PP.H4 < 0.8 PWCoCo performs conditional analyses of the summary statistics for both traits. If there is only one conditionally independent SNP in the region, this SNP will be conditioned upon. If there are multiple conditionally independent SNPs PWCoCo will isolate one SNP at the time by conditioning upon the others. Colocalization analyses are then performed on all combinations of unconditional and conditional summary statistics for the two traits. Conditional colocalization analyses were performed by conditioning the C9 GWAS on other independent genetic signals to isolate the genetic signal rs835703. PP.H4 > 0.8 PWCoCo was indicated as strong evidence for colocalization. To visualize colocalization, we generated regional association plots aligning associations with forearm fracture and fracture at any bone site with the conditional SNP associations for C9 (Fig S1).

## Results

### Genetically Predicted High Circulating C9 Levels are Causally Associated with Increased Fracture Risk in Humans

To determine the role of circulating C9 in humans, we conducted Mendelian randomization (MR) and genetic colocalization analyses. Genetically predicted higher circulating C9 levels were causally associated with increased risk of forearm fractures (Odds ratio (OR) 1.47, 95% confidence interval (CI) 1.20–1.79 per standard deviation increase in circulating C9, *P*= 1.5 × 10^−4^) and fractures at any bone site (OR 1.49, 95% CI 1.26–1.75 per standard deviation increase in circulating C9, *P*= 1.8 × 10^−6^; Table S1). In sensitivity analyses using instruments derived from GWAS summary statistics of circulating proteins measured with the independent immunoassay based Olink platform, we observed similar causal associations between circulating C9 and both forearm fractures (OR 1.46, 95% CI 1.25–1.71) and fractures at any bone site (OR 1.43, 95% CI 1.24–1.65; Table S2). Conditional colocalization analyses were performed, where the genetic signal rs835703 was isolated by conditioning the C9 GWAS on other independent genetic signals, to determine whether the genetic signals for circulating C9 levels and fracture risk originate from the same causal variant within the *C9* locus. Strong evidence for colocalization with the genetic signal for circulating C9 levels was observed for the genetic signals for forearm fractures (PP.H4 = 0.95; Fig S1A, Fig S1C, Table S3) and fractures at any bone site (PP.H4 = 0.94; Fig S1B, Fig S1C, Table S4). These findings support a causal relationship between high levels of circulating C9 and skeletal fragility in humans.

### C9 Expression is High in Liver and Low in Bone of Mice

**Fig. 1:**
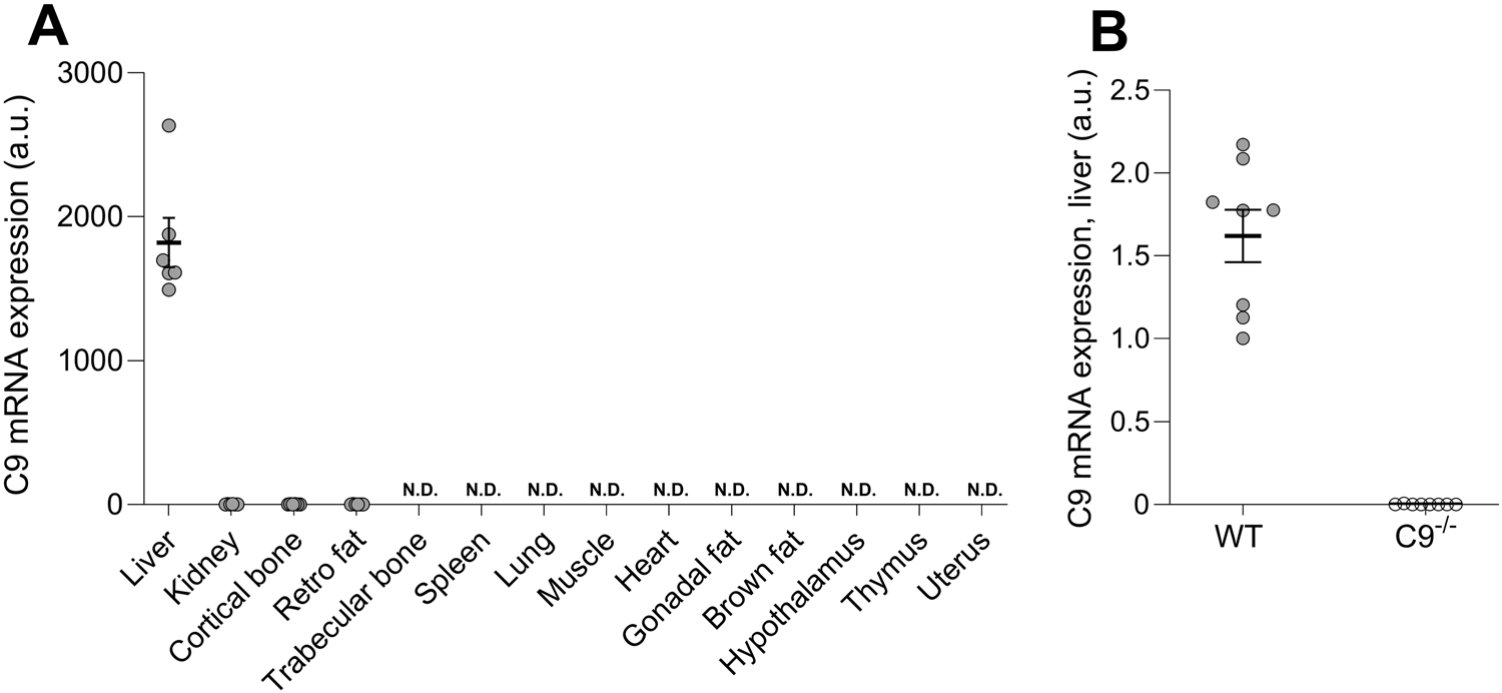
*C9* expression in liver and bone. **A** *C9* mRNA expression in liver, kidney, spleen, lung, muscle, heart, hypothalamus, thymus, uterus, retroperitoneal (retro) fat, gonadal fat, brown fat, and cortical- and trabecular bone of female WT mice. **B** *C9* mRNA expression in liver of female WT and *C9*^−/−^ mice (n = 8). Data are presented as individual values and means (horizontal lines) ± SEM. A.u., arbitrary units; N.D., not detectable

We next performed descriptive and mechanistic studies in mice. Expression analyses revealed high *C9* expression in the liver and low *C9* expression in cortical bone, whereas trabecular bone had no detectable *C9* expression (Fig. 1A). These findings demonstrate that *C9* expression is high in liver and low in bone of mice.

### *C9* Deficiency Leads to Context Dependent Effects on Vertebral Trabecular Bone in Female Mice

Mice heterozygous for global *C9* inactivation (*C9*^+/-^) were obtained from the MMRRC at University of California at Davis (see Materials and Methods [17]). The *C9*^+/-^ mice were fertile and *C9*^−/−^ offspring were viable and appeared normal. The *C9*^−/−^ mice showed complete absence of *C9* expression in the liver, confirming the validity of the knockout model (Fig. 1B). There were no significant differences in body weight or bone length between *C9*^−/−^ and WT mice (Table 1).

**Table 1:** Body weight, femur length, and vertebral height of WT and *C9* ^−/−^mice

	Females			Males		
	WT (n = 8)	*C9*^−/−^(n = 8)	*P*-value	WT (n = 7)	*C9*^−/−^(n = 8)	*P*-value
Body weight (g)	27.3 ± 1.0	24.8 ± 1.0	0.10	34.3 ± 1.1	33.6 ± 0.6	0.50
Femur length (mm)	15.9 ± 0.1	15.8 ± 0.1	0.82	15.9 ± 0.1	16.2 ± 0.2	0.84
Vertebral height (mm)	3.63 ± 0.07	3.58 ± 0.01	0.55	3.71 ± 0.06	3.68 ± 0.09	0.20

Body weight, femur length, and vertebral height of 17-week-old female and male WT and *C9*^− / −^  mice. Values are presented as mean ± SEM. Statistical analyses between *C9* ^− / −^ and WT mice were performed separately for females and males using Student’s t-test. *P*< 0.05 was considered statistically significant

**Fig. 2:**
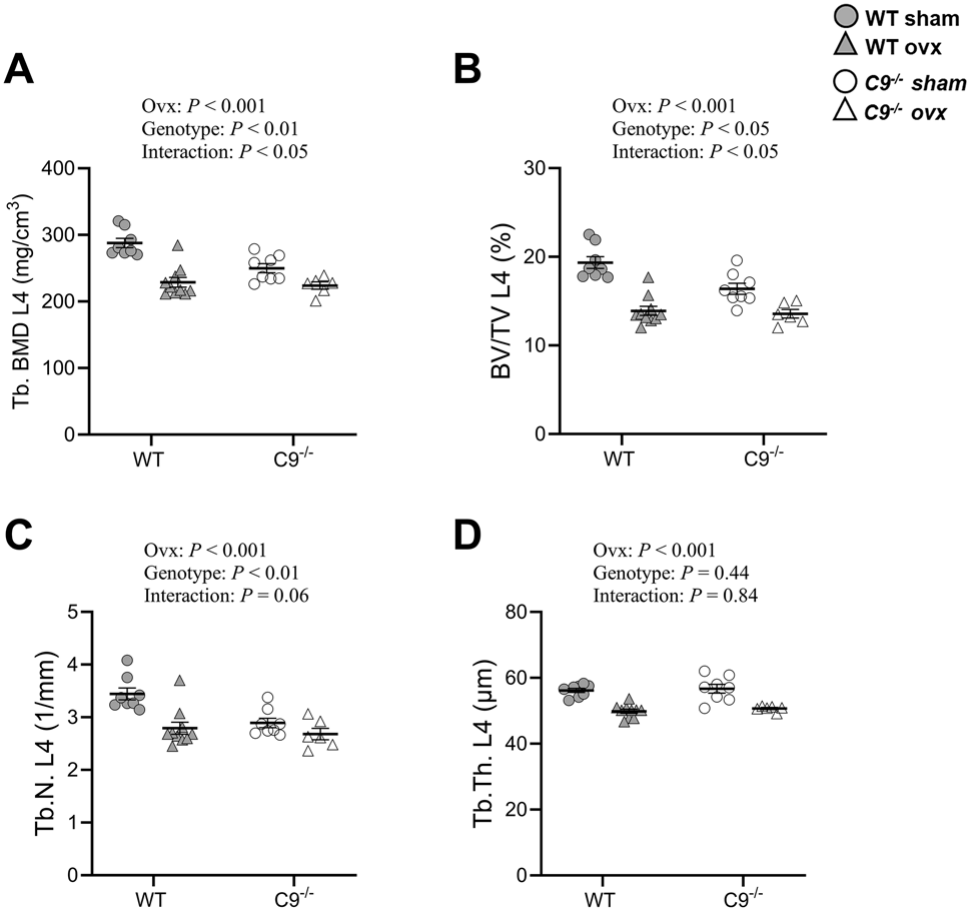
L4 vertebrae from 17-week-old sham-operated or ovariectomized (ovx) female mice (n = 6-10) were analyzed with μCT. Two-way ANOVA evaluated effects of ovx (sham vs. ovx), genotype (WT vs. *C9*^*−/−*^) and interaction (ovx-by-genotype). Trabecular **A** BMD, **B** BV/TV and **C** number were affected by both ovx and genotype, whereas **D** trabecular thickness was affected only by ovx. Significant ovx-by-genotype interactions were observed for trabecular BMD and BV/TV. Data are shown as individual values with mean (horizontal lines) ± SEM. P < 0.05 was considered statistically significant. BMD, bone mineral density; BV/TV, bone volume fraction.

The vertebral trabecular bone was affected in female mice, demonstrated by lower trabecular BMD (− 13.1 ± 2.4%, *P*< 0.005), trabecular bone volume fraction (BV/TV; − 15.3 ± 3.2%, *P*< 0.01), and trabecular number (− 16.0 ± 2.6%, *P*< 0.005), compared to WT mice (Fig. 2). Despite reduced vertebral trabecular bone mass in *C9*^−/−^ female mice, circulating bone turnover markers (CTX-I and P1NP) and histomorphometric indices of trabecular bone formation (mineralized surface/bone surface, mineral apposition rate, bone formation rate) were unchanged, suggesting a new steady state of bone turnover by 17 weeks of age (i.e. end of study; Table 2). The vertebral bone strength, measured as maximal load at failure, did not differ significantly between *C9*^−/−^ and WT mice (Table 2).

**Table 2:** Bone turnover markers and vertebral bone strength- and histomorphometry analyses in female WT and *C9*^−/−^ mice

	WT (n = 8)	*C9*^−/−^(n = 8)	*P*-value
*Bone turnover markers in serum*			
CTX-I (ng/ml)	7.86 ± 0.59	9.35 ± 1.01	0.25
P1NP (ng/ml)	40.5 ± 2.4	41.2 ± 3.2	0.87
*Vertebra*			
F_max_ (N)	24.4 ± 2.3	24.0 ± 2.5	0.90
MAR (μm/day)	1.78 ± 0.06	1.67 ± 0.22	0.22
MS/BS (%)	0.29 ± 0.01	0.29 ± 0.03	0.85
BFR/BS (μm^3^/μm^2^/day)	0.52 ± 0.04	0.48 ± 0.05	0.58

Serum levels of the bone resorption marker CTX-I and the bone formation marker P1NP were measured. Mechanical strength of L5 vertebra was assessed and dynamic histomorphometry was performed on L4 vertebra in 17-week-old sham-operated female mice. Statistical comparison between WT and *C9*^−/−^mice were performed using Student's t-test. Values are presented as mean ± SEM. *P*< 0.05 was considered statistically significant. CTX-I, collagen type I C-terminal telopeptides; P1NP, procollagen type I N-terminal propeptide; F_max_, maximal load at failure; MAR, mineral apposition rate; MS/BS, mineral surface/bone surface; BFR/BS, bone formation rate/bone surface

It has previously been shown that female mice lacking C3 are partially protected against bone loss following ovariectomy compared to WT mice [12, 13]. We, therefore, explored whether *C9*^−/−^ mice were protected against ovariectomy-induced vertebral trabecular bone loss. To ensure comparability, female mice were allocated to sham or ovariectomy groups with matched body weights within each genotype. Ovariectomy reduced the relative weight of the estrogen-sensitive uterus and increased the relative weight of the thymus in both WT and *C9*^−/−^ mice (Table 3). No significant effects of genotype or ovariectomy by genotype interaction were observed for any of these effects. As expected, ovariectomy reduced vertebral trabecular bone parameters, including volumetric BMD, BV/TV, trabecular number and trabecular thickness (Fig. 2A-D). Notably, *C9*^−/−^ mice were partially protected against ovariectomy-induced bone loss, demonstrated by a significant ovariectomy by genotype interaction for vertebral trabecular BMD and BV/TV (Fig. 2A-B). A significant genotype-by-ovariectomy interaction was observed for vertebral trabecular BMD (F(1,28) = 5.31, *P*= 0.029). Holm–Šidák–adjusted pairwise post hoc analyses showed that ovariectomy reduced trabecular BMD in WT mice (− 20.5 ± 7.9%, *P*< 0.0001), whereas *C9*^−/−^ mice showed a smaller and statistically non‑significant reduction (− 10.4 ± 5.3%, *P*= 0.063). Under sham conditions, *C9*^−/−^ mice had lower trabecular BMD than WT (− 13.1 ± 6.7%, *P*= 0.0028), but this genotype difference was abolished after ovariectomy (− 2.1 ± 5.8%, *P*= 0.63). These findings demonstrate that ovariectomy‑induced BMD loss was more pronounced in WT mice than in *C9*^−/−^ mice. A similar pattern was observed for trabecular BV/TV, where two‑way ANOVA also detected a significant genotype-by-ovariectomy interaction (F(1,28) = 4.81, *P*= 0.037). Ovariectomy reduced trabecular BV/TV in both genotypes, but to a greater extent in WT mice (WT: − 28.2 ± 8.4%, *P*< 0.0001; *C9*^−/−^:− 17.2 ± 7.3%, *P*= 0.024). Under sham conditions, *C9*^−/−^ mice exhibited lower trabecular BV/TV than WT (− 15.3 ± 9.2%, *P*= 0.0085), whereas no genotype difference was present after ovariectomy (− 2.3 ± 8.6%, *P*= 0.999). Thus, ovariectomy abolished the baseline genotype difference and induced a more pronounced reduction in WT mice compared with *C9*^−/−^ mice, thereby explaining the significant genotype-by-ovariectomy interaction identified in the two-way ANOVA analyses (Fig. 2A–B). Female *C9*^−/−^ mice were not protected against ovariectomy-induced bone loss in femur (Table 4). No skeletal phenotype was observed in male *C9*^−/−^ mice (Table S5). Orchidectomy resulted in the expected reductions in weights of androgen responsive tissues (vesicle seminalis and levator ani muscle; Table S6) and reduced trabecular and cortical bone mass in male mice (Table S5). No significant effect of genotype was observed. These findings indicate that male *C9*^−/−^ mice are not protected against orchidectomy-induced bone loss.

**Table 3:** Relative tissue weights in sham-operated and ovx WT and *C9* ^−/−^ female mice

	WT		*C9*^−/−^		*P*-value		
	Sham (n = 8)	Ovx (n = 10)	Sham (n = 8)	Ovx (n = 6)	Ovx	Genotype	Interaction
Thymus/BW (%)	0.28 ± 0.02	0.37 ± 0.02	0.31 ± 0.01	0.39 ± 0.02	< 0.001	0.44	0.48
Uterus/BW (%)	0.26 ± 0.04	0.06 ± 0.01	0.33 ± 0.08	0.06 ± 0.01	< 0.001	0.18	0.97
Gonadal fat/BW (%)	4.24 ± 0.30	4.34 ± 0.22	3.48 ± 0.46	3.78 ± 0.49	0.51	0.06	0.86
Liver/BW (%)	4.45 ± 0.08	4.30 ± 0.15	4.42 ± 0.08	4.32 ± 0.12	0.30	0.97	0.84
Spleen/BW (%)	0.34 ± 0.03	0.39 ± 0.02	0.43 ± 0.06	0.35 ± 0.01	0.62	0.40	0.09

Tissue weights were measured in 17-week-old sham-operated and ovariectomized (ovx) female mice. A two-way ANOVA was used to evaluate the effects of ovx (sham vs. ovx), genotype (WT vs. *C9*^−/−^), and their interaction (ovx by genotype). Values are presented as mean ± SEM. *P*< 0.05 was considered statistically significant. BW, body weight

**Table 4:** Trabecular and cortical bone parameters in femur in female WT and *C9*^−/−^ mice

	WT		*C9*^−/−^		*P*-value		
	Sham (n = 8)	Ovx (n = 10)	Sham (n = 8)	Ovx (n = 6)	Ovx	Genotype	Interaction
Trabecular BMD (mg/cm^3^)	248 ± 12	193 ± 12	256 ± 20	184 ± 9	< 0.001	0.96	0.57
Cortical thickness (mm)	0.205 ± 0.002	0.186 ± 0.003	0.201 ± 0.004	0.185 ± 0.002	< 0.001	0.53	0.61
Cortical area (mm^2^)	0.93 ± 0.02	0.84 ± 0.01	0.89 ± 0.02	0.83 ± 0.01	< 0.001	0.18	0.52

Femur from 17-week-old sham-operated and ovariectomized (ovx) female mice were analyzed by pQCT. Two-way ANOVA was used to evaluate the effects of ovx (sham vs. ovx), genotype (WT vs. *C9*^−/−^), and their interaction (ovx by genotype). Values are presented as mean ± SEM. *P*< 0.05 was considered statistically significant. BMD, bone mineral density

## Discussion

While other complement components have been associated with bone mass regulation, the role of C9 was unknown. In this study, we demonstrate that genetically predicted higher circulating *C9* levels were causally associated with higher risk of fractures in humans. Expression analyses in mice revealed that *C9* is mainly expressed in the liver, although low levels were also detected in cortical bone. Adult female mice with global C9 deletion exhibited lower vertebral trabecular bone volume fraction, but they were partly protected against ovariectomy-induced vertebral trabecular bone loss. The impact of C9 on bone health may vary depending on the species studied, the distinction between circulating and local C9, and the specific physiological or pathological context.

MR is a method of causal inference that uses genetic variants as instrumental variables to test the role of exposures in disease outcomes. MR should be complemented with colocalization analyses to exclude confounding by linkage disequilibrium [29]. Importantly, the observed causal associations between genetically predicted circulating C9 levels and fracture risk were supported by colocalization analyses, demonstrating that the genetic signals for circulating C9 levels and fracture risk arise from the same causal variant within the C9 locus. In sensitivity analyses using instruments derived from GWAS summary statistics of circulating proteins measured with the independent immunoassay-based Olink platform, we observed similar causal associations between circulating C9 and fractures. A limitation of our Mendelian randomization analysis is that the instrument consisted of a single SNP. The use of a single genetic instrument precludes the application of sensitivity analyses to assess horizontal pleiotropy and may limit the robustness of causal inference. Together these findings indicate that high levels of circulating, mainly liver derived, C9 increase fracture risk in humans.

Our subsequent mechanistic studies in mice demonstrate that mice with global C9 deficiency exhibit sex-specific skeletal changes, suggesting a role of C9 in bone homeostasis that differ between males and females. Female C9-deficient mice, but not males, exhibited markedly reduced vertebral trabecular bone loss compared with WT mice. Despite this reduction in axial trabecular bone mass, circulating bone turnover markers (CTX-I and P1NP) and histomorphometric indices of trabecular bone formation (mineralized surface/bone surface, mineral apposition rate, and bone formation rate) were unchanged, suggesting that a new steady state of bone turnover had been reached. This apparent paradox, reduced baseline bone mass yet partial protection against ovariectomy induced loss, suggests that C9 contributes both to the establishment of normal estrogen dependent bone homeostasis and to the resorptive response triggered by estrogen withdrawal. This finding indicates that C9 is critical for maintaining axial trabecular bone in gonadally intact females, but not males, suggesting a potential interaction between C9 and sex steroids. The sex‑specific nature of the phenotype suggests that complement components may modulate the estrogen responsiveness of bone or immune cells. Estrogen can influence complement activity by regulating immune‑cell transcriptional programs through estrogen‑receptor signaling, which affects expression of complement‑related genes and innate‑immune pathways [30]. These interactions offer a plausible mechanism for the sex‑specific phenotype observed here, suggesting that loss of C9 may disrupt an estrogen‑dependent complement pathway that influences bone turnover.

This notion is supported by our observation that C9-deficient female mice were partially protected against ovariectomy-induced bone loss. Thus, the bone‑sparing effect of endogenous estrogens may depend in part on intact C9 signaling, offering a unified explanation for both the reduced baseline vertebral bone mass and the attenuated loss after ovariectomy.

Male *C9*^−/−^ mice exhibited no skeletal phenotype, which may reflect lower basal complement activation and the comparatively limited influence of androgens on complement‑related transcriptional pathways [31]. Unlike estrogens, which can directly modulate complement‑linked immune signaling, androgen effects appear mainly immunosuppressive and less targeted, potentially rendering male bone less sensitive to C9 deficiency.

C9 deficiency resulted in an axially restricted trabecular phenotype, with alterations present in the vertebrae but not the femora. Although the underlying mechanism is uncertain, site specific differences in the regulation of trabecular microarchitecture exist [32], which may increase axial sensitivity to systemic influences such as complement related changes.

Previous studies on mice lacking CD59, the membrane-bound inhibitor of the MAC, have reported inconsistent skeletal phenotypes. One study found increased femoral cortical and trabecular bone volume in male CD59-deficient mice at 8 weeks of age, along with reduced cortical BMD in both sexes [15]. At 20 weeks of age, CD59-deficient male mice continued to exhibit increased cortical bone volume and reduced BMD, whereas at 50 weeks of age cortical BMD was increased in both sexes compared to WT mice [15]. In contrast, another study reported no skeletal changes in 17-week-old CD59-deficient males [14]. Unlike CD59 deficiency, which allows the formation of the C9 lytic pore of the MAC and potential autologous cell damage, C9 deficiency prevents MAC completion. One could easily hypothesize that mice deficient of C9 would have opposite skeletal phenotypes compared to mice deficient of CD59. However, the female C9-deficient mice have a decreased vertebral bone volume, whereas the males have no skeletal phenotype. These findings indicate that C9 deficiency does not simply produce opposing skeletal outcomes from CD59 deficiency but rather highlight distinct and context dependent roles of individual MAC components in bone homeostasis.

The present study demonstrates that C9 deficiency in mice is non-lethal and that C9-deficient mice appear healthy, which aligns with clinical observations. Although complete C9 deficiency has not been extensively characterized in humans, available data indicate that it is similarly non-lethal [33]. While rare globally, the prevalence of C9 deficiency in Japan is approximately 0.12% [33]. Individuals lacking C9 appear clinically healthy but have increased susceptibility to meningococcal infection, albeit with lower associated mortality compared to immunocompetent individuals [33]. To date, no published data exists on skeletal parameters in C9-deficient human populations.

Human studies in the US-based MrOS cohort have associated higher circulating levels of complement components 7 and 9 with accelerated BMD loss [11]. Conversely, lower concentrations of C7 and C9 have been linked to better overall health and longevity [11, 34]. Because these previous investigations were observational, they could not establish causality. Our study extends these findings by demonstrating that high circulating C9 levels are causally linked to increased fracture risk. Elevated C9 levels may reflect an upregulated acute-phase response, which supports tissue repair but, when dysregulated, may promote chronic inflammation and bone loss [35]. Our previous work support this hypothesis, showing that activation of the acute phase pathway is linked to increased hip fracture risk [36]. The acute phase response plays a critical role in maintaining tissue homeostasis in the musculoskeletal system by facilitating repair of microinjuries incurred during normal daily activity. However, dysregulation of this response may lead to a chronic inflammatory state, promoting degeneration of musculoskeletal tissues and ultimately contributing to the development of osteoporosis [37, 38]. Thus, elevated circulating levels of C9 may reflect an upregulated acute‑phase response, which could, when dysregulated, promote bone loss and increase fracture susceptibility, but should not be interpreted as a stand‑alone clinical biomarker of fracture risk. Given the skeletal effects observed in C9‑deficient mice, we note that complement‑targeted therapies involving C9 inhibition could theoretically influence bone health, although current evidence is insufficient and dedicated safety studies would be required to assess such risks.

Emerging mechanobiology studies further support a link between membrane integrity and osteocyte function [39]. Osteocytes rely on intact plasma membranes and tightly regulated Ca^2^⁺ signaling to sense mechanical load, and disuse conditions such as simulated microgravity markedly disrupt these Ca^2^⁺ dynamics [40]. Given that MAC forms stable pores capable of altering ionic flux, complement activation may impair osteocyte mechanotransduction by disturbing membrane stability and downstream Ca^2^⁺‑dependent signaling.

Limitations of the study include the absence of mechanistic insight into how estrogen interacts with complement pathways in bone, and the lack of functional assays assessing complement activation within the bone microenvironment, which should be addressed in future investigations.

In conclusion, high genetically determined circulating *C9* is causally linked to increased fracture risk in humans. In mice, global inactivation of *C9* affects vertebral trabecular bone in a sex specific and context dependent manner.

## Supplementary Information

Below is the link to the electronic supplementary material.

Supplementary file1 (DOCX 43 KB)

Supplementary file2 (XLSX 447 KB)

Supplementary file3
