# Supplementary material for: Role of Complement Component 9 in Bone Health: Causal Evidence in Humans and Mechanistic Studies in Mice
Source: Calcif Tissue Int. 2026 Apr 2;117(1):54. doi: 10.1007/s00223-026-01515-9 (PMC13046653; doi:10.1007/s00223-026-01515-9)
Supplement: Supplementary file 1 — Supplementary file1 (DOCX 43 KB) [file 223_2026_1515_MOESM1_ESM.docx]

**Table S1. Associations for the *C9* cis-pQTL rs835703 with circulating C9, forearm fractures and fractures at any bone site.**

| **Trait** | **EA** | **OA** | **EAF** | **Beta** | **SE** | ***P*-value** |
| --- | --- | --- | --- | --- | --- | --- |
| Circulating C9 | G | C | 0.61 | 0.080 | 0.01 | 6.8E-24 |
| Forearm fracture | G | C | 0.61 | 0.031 | 0.01 | 1.5E-04 |
| Any fracture | G | C | 0.60 | 0.032 | 0.01 | 1.8E-06 |

The associations for rs835703 with these traits were derived from previously published publicly available GWAS summary statistics for circulating proteins [1], forearm fracture [2], and fracture at any bone site [3]. EA, effect allele; OA, other allele; EAF, effect allele frequency; SE, standard error.

**Table S2. Associations for the *C9* cis-pQTL rs700233 with circulating C9, forearm fractures and fractures at any bone site.**

| **Trait** | **EA** | **OA** | **EAF** | **Beta** | **SE** | ***P*-value** |
| --- | --- | --- | --- | --- | --- | --- |
| Circulating C9 | G | A | 0.60 | 0.09 | 0.01 | 2.3E-41 |
| Forearm fracture | G | A | 0.61 | 0.03 | 0.01 | 1.9E-06 |
| Any fracture | G | A | 0.60 | 0.03 | 0.01 | 8.9E-07 |

The associations for rs700233 with these traits were derived from previously published publicly available GWAS summary statistics for circulating proteins [1], forearm fracture [2], and fracture at any bone site [3]. EA, effect allele; OA, other allele; EAF, effect allele frequency; SE, standard error.

**Table S5. Trabecular and cortical bone parameters of vertebra and femur in male WT and *C9^−/−^* mice.**

|  | ***WT*** | | ***C9^-/-^*** | | ***P*-value** | | |
| --- | --- | --- | --- | --- | --- | --- | --- |
|  | **Sham** (n = 7) | **Orx** (n = 9) | **Sham** (n = 7) | **Orx** (n = 8) | **Orx** | **Genotype** | **Interaction** |
| *L4 vertebra* |  |  |  |  |  |  |  |
| Trab. BMD (mg/cm^3^) | 298 ± 12 | 197 ± 5 | 288 ± 5 | 194 ± 7 | < 0.001 | 0.40 | 0.65 |
| BV/TV (%) | 19.0 ± 1.2 | 10.5 ± 0.4 | 18.4 ± 0.4 | 10.7 ± 0.6 | < 0.001 | 0.78 | 0.62 |
| Trab. number (1/mm) | 3.92 ± 0.19 | 2.17 ± 0.06 | 3.57 ± 0.10 | 2.19 ± 0.09 | < 0.001 | 0.16 | 0.12 |
| Trab. thickness (mm) | 0.048 ± 0.001 | 0.048 ± 0.001 | 0.052 ± 0.001 | 0.049 ± 0.001 | 0.26 | 0.15 | 0.19 |
| *Femur* |  |  |  |  |  |  |  |
| Trab. BMD (mg/cm^3^) | 264 ± 21 | 189 ± 6 | 317 ± 12 | 182 ± 9 | < 0.001 | 0.09 | < 0.05 |
| Cortical thickness (mm) | 0.191 ± 0.004 | 0.162 ± 0.003 | 0.188 ± 0.003 | 0.161 ± 0.005 | < 0.001 | 0.64 | 0.68 |
| Cortical area (mm^2^) | 0.99 ± 0.03 | 0.84 ± 0.01 | 0.99 ± 0.02 | 0.84 ± 0.02 | < 0.001 | 0.86 | 0.99 |

Bone parameters of L4 and femur were analyzed for 17-week-old sham-operated or orchidectomized (orx) male mice. A two-way ANOVA was used to evaluate the effect of orx (sham *vs*. orx), genotype (WT *vs*. *C9^−/−^*) and interaction (orx by genotype). Values are presented as mean ± SEM *P* < 0.05 was considered statistically significant. BMD, bone mineral density; BV/TV, bone volume/tissue volume.

**Table S6. Relative tissue weights in sham-operated and orx WT and *C9^−/−^* male mice.**

|  | ***WT*** | | ***C9^-/-^*** | | ***P*-value** | | |
| --- | --- | --- | --- | --- | --- | --- | --- |
|  | **Sham** (n = 7) | **Orx** (n = 9) | **Sham** (n = 7) | **Orx** (n = 8) | **Orx** | **Genotype** | **Interaction** |
| Thymus/BW (%) | 0.19 ± 0.02 | 0.38 ± 0.02 | 0.20 ± 0.01 | 0.37 ± 0.01 | < 0.001 | 0.86 | 0.46 |
| Vesicle seminalis/BW (%) | 0.89 ± 0.05 | 0.19 ± 0.07 | 0.93 ± 0.05 | 0.09 ± 0.01 | < 0.001 | 0.86 | 0.07 |
| Levator ani/BW (%) | 0.23 ± 0.02 | 0.10 ± 0.01 | 0.25 ± 0.01 | 0.11 ± 0.01 | < 0.001 | 0.66 | 0.86 |
| Gonadal fat/BW (%) | 4.09 ± 0.54 | 3.19 ± 0.58 | 3.10 ± 0.53 | 2.73 ± 0.42 | 0.24 | 0.18 | 0.62 |
| Liver/BW (%) | 4.50 ± 0.09 | 4.51 ± 0.19 | 4.67 ± 0.12 | 4.27 ± 0.13 | 0.20 | 0.82 | 0.18 |
| Spleen/BW (%) | 0.30 ± 0.01 | 0.39 ± 0.01 | 0.32 ± 0.03 | 0.38 ± 0.02 | < 0.001 | 0.69 | 0.61 |

Tissue weights were measured in 17-week-old sham-operated or orchidectomized (orx) male mice. A two-way ANOVA was used to evaluate the effects of orx (sham *vs*. orx), genotype (WT *vs*. *C9^−/−^*), and interaction (orx by genotype). Values are presented as mean ± SEM. *P* < 0.05 was considered statistically significant. BW, body weight.

**References**

1. Eldjarn GH, Ferkingstad E, Lund SH, Helgason H, Magnusson OT, Gunnarsdottir K, et al. Large-scale plasma proteomics comparisons through genetics and disease associations. Nature. 2023;622(7982):348-58.

2. Nethander M, Moverare-Skrtic S, Kampe A, Coward E, Reimann E, Grahnemo L, et al. An atlas of genetic determinants of forearm fracture. Nat Genet. 2023;55(11):1820-30.

3. Morris JA, Kemp JP, Youlten SE, Laurent L, Logan JG, Chai RC, et al. An atlas of genetic influences on osteoporosis in humans and mice. Nat Genet. 2019;51(2):258-66.
