# Supplementary material for: Role of Complement Component 9 in Bone Health: Causal Evidence in Humans and Mechanistic Studies in Mice
Source: Calcif Tissue Int. 2026 Apr 2;117(1):54. doi: 10.1007/s00223-026-01515-9 (PMC13046653; doi:10.1007/s00223-026-01515-9)
Supplement: Supplementary file 3 — Supplementary file3 [file 223_2026_1515_MOESM3_ESM.pdf]

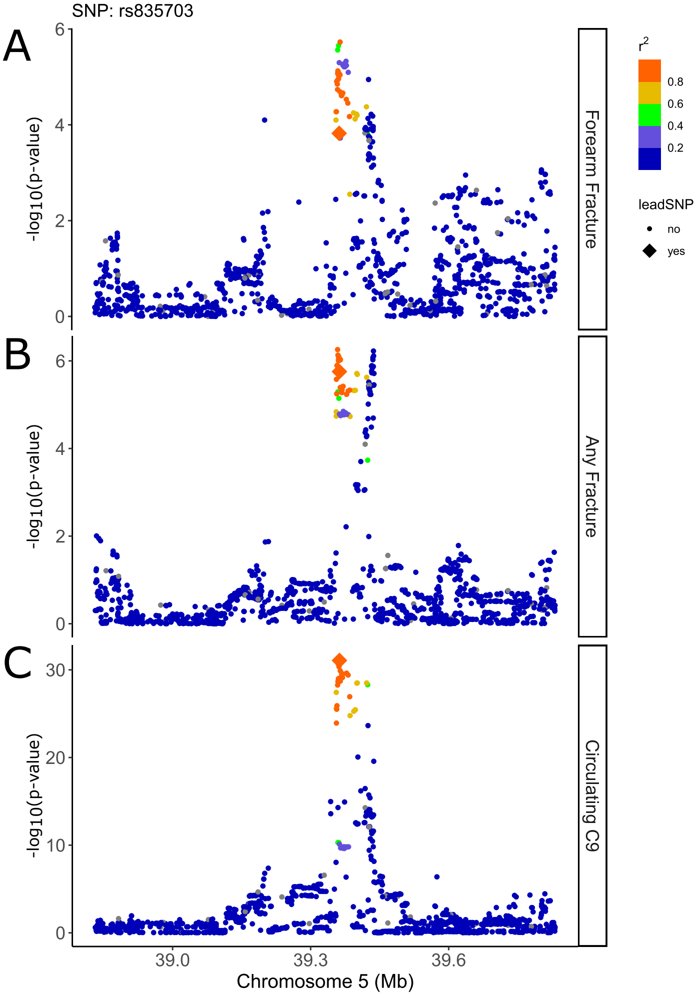

Supplementary Figure 1: Regional association plots for A: forearm fracture GWAS, B: fracture at any bone site GWAS and C: conditional GWAS for circulating C9 in the colocalized C9 region. The C9 GWAS was conditioned on rs10512702, rs261753 and rs358501. The lead SNP, rs835703, is marked as a diamond and the color scale represents the linkage with the lead SNP.
